# Comprehensive evaluation model of ecotourism, economic development and ecological environment-taking Shennongjia National Park as an example

**DOI:** 10.3389/fpubh.2025.1569684

**Published:** 2025-04-30

**Authors:** Xin Cui

**Affiliations:** Zhoukou Polytechnic, College of Culture and Education, Zhoukou, Henan, China

**Keywords:** national parks, ecotourism, economic development, ecological stress, coupling coordination degree, spatial econometric modeling

## Abstract

**Introduction:**

This study investigates the interaction between ecotourism and economic development, and their impact on the ecological environment within national parks. Shennongjia National Park serves as a case study for this analysis.

**Methods:**

A comprehensive evaluation model was constructed, comprising the Ecotourism Development Index (ETI), Economic Development Index (EDI), and Ecological Pressure Index (EPI). The coupling coordination degree model (D) and the Spatial Durbin Model (SDM) were employed to quantify synergistic effects and spatial interaction mechanisms among these systems. Multi-source data from 2016 to 2022 were utilized.

**Results:**

Ecotourism development exhibits a significant positive impact on regional economic growth (elasticity coefficient 0.68, *p* < 0.01). However, increased tourist numbers correlate with heightened ecological pressure, notably vegetation degradation and water quality decline in core scenic areas (e.g., Shennongding). Spatial analysis reveals significant positive correlations among ecotourism, economic growth, and ecological pressure (global Moran’s Index), with “high-high” and “low-low” clustering patterns. Local Indicators of Spatial Association (LISA) identify Muyu Town as a high-value cluster and Xiagu Township as a low-value cluster. The coupling coordination degree improved from near imbalance (*D* = 0.48) in 2016 to intermediate coordination ((*D* = 0.75) in 2022. A prominent ecological-environmental lagging contradiction (ETI > EPI) is observed, attributed to tourist overload and insufficient ecological restoration funding.

**Discussion:**

The findings indicate that while ecotourism significantly drives economic growth, it also increases ecological pressure. Spatial dependencies highlight the need for regional coordination in development strategies. Addressing the ecological-environmental lag, particularly through managing tourist capacity and increasing investment in ecological restoration, is crucial for achieving sustainable development. The study proposes development pathways and strategies to promote the coordinated advancement of ecotourism, economic growth, and ecological environmental protection.

## 1 Introduction

The core concept of ecotourism is to protect the natural environment through tourism activities while improving the quality of life of local residents (1). Ecotourism is based on the natural environment, emphasizes extensive stakeholder participation, and achieves the dual goals of conservation and development through strict planning management and regulatory frameworks (2). This form of tourism not only benefits the natural environment, communities, tourism enterprises and tourists, but also promotes sustainable environmental, social and economic development (3). Since its introduction to China in the last century, ecotourism has rapidly become one of the fastest growing areas of tourism. With increasing public awareness, more and more nature reserves have become popular tourist destinations (4).

China’s protected nature areas are mainly categorized into three types: national parks, nature reserves and nature parks (5). Among them, national parks are approved and established by the state, aiming to protect nationally representative natural ecosystems while realizing scientific protection and rational utilization of natural resources (6). As the most important and biodiversity-rich areas of China’s natural ecosystems, the construction of national parks has been vigorously promoted in recent years. The General Program for the Establishment of the National Park System, released in 2017, clearly states that the core function of national parks is to protect the originality and integrity of the important natural ecosystems, and at the same time, it also has the comprehensive functions of scientific research, education, and recreation, etc. (7).

National parks are not only important areas for the protection of natural ecosystems, but also the core carrier for the inheritance of natural and cultural values (8). With the increasing awareness of ecological protection worldwide, ecotourism has gradually become an important model for the development of national parks and their surrounding areas (3). Through the rational use of natural resources, ecotourism provides tourists with the opportunity to get close to nature and understand nature, and at the same time creates economic benefits for local communities and promotes the sustainable development of the regional economy (9).

However, the contradiction between economic development and ecological protection has become increasingly prominent. National parks shoulder the important mission of protecting rare species, maintaining ecological balance and preserving natural heritage (10). At the same time, the development needs of the neighboring communities and the potential of the tourism market make it a key issue to balance ecotourism and economic development (9). On the one hand, ecotourism can drive the development of related industries such as transportation, catering, and accommodation, create employment opportunities, and increase the income of residents, thus promoting regional economic prosperity (11). On the other hand, excessive or irrational tourism development may cause negative impacts on the ecological environment, such as the destruction of vegetation cover, pollution of water sources, and disturbance of wildlife habitats, which may in turn threaten the stability of the ecosystem (12).

In recent years, with the rapid development of global tourism and the increased demand for natural ecological experiences, ecotourism in national parks has shown a booming trend. However, the issue of the relationship between ecotourism and economic development and its pressure on the ecological environment has also attracted extensive attention from academics, governmental departments and the community. An in-depth study of the interaction mechanism between ecotourism and economic development in national parks and an accurate assessment of the impact of ecotourism on the ecological environment are of great significance for the formulation of scientific management policies and the realization of sustainable development of ecotourism. For this reason, this study developed a comprehensive evaluation model using a multidimensional system and spatial measurement methods. It aims to analyze the interaction between ecotourism and economic development in Shennongjia National Park through theoretical modeling and empirical analysis. The findings provide a scientific basis for the sustainable development of global nature reserves.

This study focuses on Shennongjia National Park, a biodiversity hotspot in China, to systematically analyze the interaction between ecotourism, economic development, and ecological environment. Specifically, the study aims to: (1) quantify the economic driving effect of ecotourism on regional economic growth; (2) assess the ecological pressure caused by tourism activities and its spatial distribution; (3) analyze the coupling coordination degree between ecotourism, economic development, and ecological environment; and (4) propose sustainable development strategies for national parks based on empirical findings.

To achieve these objectives, the study employs a comprehensive theoretical framework that integrates three key models: (1) the Comprehensive Evaluation Model, which comprises three indices—Ecotourism Development Index (ETI), Economic Development Index (EDI), and Ecological Pressure Index (EPI)—to assess the interactions among these systems in national parks; (2) the Coupling Coordination Degree Model, which quantifies the synergistic states and dynamic evolution laws of ecological protection and tourism development; and (3) the Spatial Durbin Model (SDM), which analyzes the spatial interaction mechanisms among ecotourism, economic development, and ecological pressure, providing insights into spatial spillover effects and dependencies. By integrating these models, the study provides a robust framework for understanding the complex relationships between ecotourism, economic growth, and ecological conservation in national parks.

## 2 Literature review

### 2.1 Overview of national parks at home and abroad

National parks, with their unique natural landscapes and rich biodiversity, have become globally recognized tourist destinations. They are not only ideal places for nature education, recreation, scientific research and eco-tourism, but also an important carrier for the protection of natural ecosystems. National parks around the world are distinctive and have a long historical origin. For example, the Vatnajökull National Park in Iceland is famous for its unique landscape of glaciers, volcanoes, canyons, forests and waterfalls, and is known as the “Land of Ice and Fire” (13). The Lake District National Park in the United Kingdom is known for its magnificent lakes, mountains, and nature reserves, and is known as one of the most beautiful areas in England, as well as a popular vacation destination (14). Russia’s Białoyeża National Forest Park is rich in flora and fauna, one of the few pristine forests in the world, and an important wildlife habitat (15). The Lauterbrunnen Valley National Park in Switzerland is known for its spectacular waterfall scenery and is the largest nature reserve in Switzerland (16). Mount Olympus National Park in Greece, on the other hand, is known for its biodiversity and cultural heritage, and is an example of the perfect combination of nature and culture (17).

In China, the construction of national parks has made remarkable progress in recent years. In 2017, the release of the General Program for the Establishment of the National Park System marked the formal establishment of China’s national park system. Currently, China has established several pilot national parks, including Sanjiangyuan National Park (18), Giant Panda National Park (19), Northeast Tiger and Leopard National Park (20), and Shennongjia National Park (21). These national parks not only protect the most representative natural ecosystems in China, but also have scientific research, education and recreational functions.

The Sanjiangyuan National Park, for example, is located in the hinterland of the Qinghai-Tibetan Plateau, the birthplace of the Yangtze, Yellow and Lancang Rivers, and is known as the “Water Tower of China.” It has a unique alpine ecosystem and rich biodiversity, and is an important habitat for rare species such as the snow leopard and Tibetan antelope. The Giant Panda National Park, which spans Sichuan, Shaanxi and Gansu provinces, aims to protect the giant panda and its habitat while promoting regional ecotourism and community development. The Northeast Tiger and Leopard National Park, located in Jilin and Heilongjiang Provinces, is the main habitat for China’s northeast tigers and leopards, and is important for the recovery of endangered species populations. Shennongjia National Park, on the other hand, is known for its rich flora and fauna and unique ecosystems, and serves as a natural laboratory for the study of biodiversity and ecological conservation.

### 2.2 Ecotourism

Ecotourism is defined as a form of tourism where visitors travel to relatively undisturbed natural areas to appreciate and learn about the natural environment, wildlife, and cultural heritage, while promoting the conservation of these resources and improving the well-being of local communities. This definition draws from the seminal work of Ceballos Lascurain (1983), who first formally introduced the concept of ecotourism as a travel activity focused on exploring and appreciating natural areas (22). The International Ecotourism Society further elaborates that ecotourism should be responsible, preserving ecological and cultural integrity while enhancing the living standards of local residents. Additionally, the International Union for Conservation of Nature (IUCN) emphasizes that ecotourism should contribute to the conservation of biodiversity and the sustainable use of natural resources (23).

In 1992, the concept of “ecotourism” was introduced into China, which attracted many scholars to conduct in-depth research. Zhang et al. (24) defined ecotourism as a tourism system in which tourists can achieve sustainable development by exploring nature and obtaining physical and mental satisfaction during leisure vacations (3). Wan et al. (25) pointed out that ecotourism not only raises environmental awareness among tourists and local communities, but also supports environmental protection while promoting economic development. By mobilizing the community, ecotourism can achieve sustainable and healthy development. According to Xu et al. (26), the object of ecotourism includes not only natural ecosystems, but also human ecosystems in natural areas. In the process of ecotourism development, all stakeholders should have a sense of responsibility for the protection of natural resources and sustainable development, and the goal of environmental education should be realized in the activities. Peng et al. (27) emphasized that the rational use of high-quality tourism resources in national parks to develop tourism can both promote local economic development and give full play to the comprehensive functions of national parks. In addition, the establishment of an eco-visitor system and the advocacy of eco-civilized tourism are the inevitable requirements for eco-tourism to achieve sustainable development. The studies of these scholars have profoundly elaborated the necessity and importance of developing ecotourism in national parks.

### 2.3 Economic effects of ecotourism in national parks and its pressure on the ecological environment

Ecotourism, as a form of nature-based tourism, is regarded as a green industry that promotes regional economic development. According to the United Nations World Tourism Organization (UNWTO), the size of the global ecotourism market has grown from US$50 billion in 2010 to US$200 billion in 2022, with an average annual growth rate of more than 10%. As the main carrier of ecotourism, the economic effect of national parks is mainly reflected in the following aspects:

Direct economic contribution: through ticket revenues, tourism services and merchandise sales, ecotourism directly injects money into the local economy. For example, the U.S. national park system contributes more than $40 billion U.S. dollars to the U.S. economy every year.

Indirect economic pull: ecotourism drives the development of industries such as catering, accommodation and transportation in the surrounding communities, indirectly contributing to regional economic growth. For example, the Kruger Park in South Africa creates more than 100,000 jobs for the neighboring communities every year.

Transformation of ecological product value: Through mechanisms such as ecological compensation and carbon sink trading, ecological resources are transformed into economic value. For example, Costa Rica earns more than US$100 million annually through carbon sink trading in its national parks.

However, there are significant regional differences in the economic effects of ecotourism. In developed countries, the economic benefits of ecotourism and ecological protection can achieve a better balance due to sound management mechanisms and advanced technical means. In developing countries, however, ecotourism often leads to “over-exploitation” and “resource depletion” due to the limitations of capital, technology and management capacity.

Although ecotourism is considered an environmentally friendly form of tourism, its negative impact on the ecological environment cannot be ignored. According to the IUCN study, the environmental pressure of ecotourism is mainly reflected in the following aspects:

Habitat destruction: tourist activities may lead to vegetation trampling, soil compaction and wildlife habitat loss. For example, tourist activities in Kenya’s Masai Mara National Park have resulted in degradation of more than 20% of the grassland area.

Biodiversity decline: visitor disturbance can lead to changes in wildlife behavior and population declines. For example, visitor activities in Australia’s Great Barrier Reef National Park have resulted in a 30% decline in coral reef cover.

Environmental pollution: ecological pollution is caused by garbage, wastewater and noise generated by tourists. For example, tourist activities in Sagarmatha National Park in Nepal led to deterioration of water quality in localized areas.

Ecotourism has brought some pressure on the ecological environment while promoting economic development. How to balance the economic effect and ecological protection is an important issue to realize the sustainable development of national parks.

## 3 Methodology

This study aims to systematically assess the interactions between ecotourism, economic development and ecological environment in Shennongjia National Park and their spatial effects. To achieve this goal, this paper adopts a research framework that combines a multidimensional comprehensive evaluation model with spatial measurement methods. First, based on multi-source data, a multi-level evaluation system is constructed through the objective assignment of entropy weight method. Second, in order to assess the interaction between the three, the coupling coordination degree model is introduced to quantify the synergistic state of ecological protection and tourism development, classify the type of coupling coordination, and reveal its dynamic evolution law. In addition, to address the spatial heterogeneity problem, the Spatial Durbin Model (SDM) is used to analyze the spatial spillover effect of ecotourism on economic growth and the spatial dependence of ecological and environmental pressures by combining the geographic adjacency matrix and the distance weight matrix.

### 3.1 Overview of the study area

**Figure 1:**
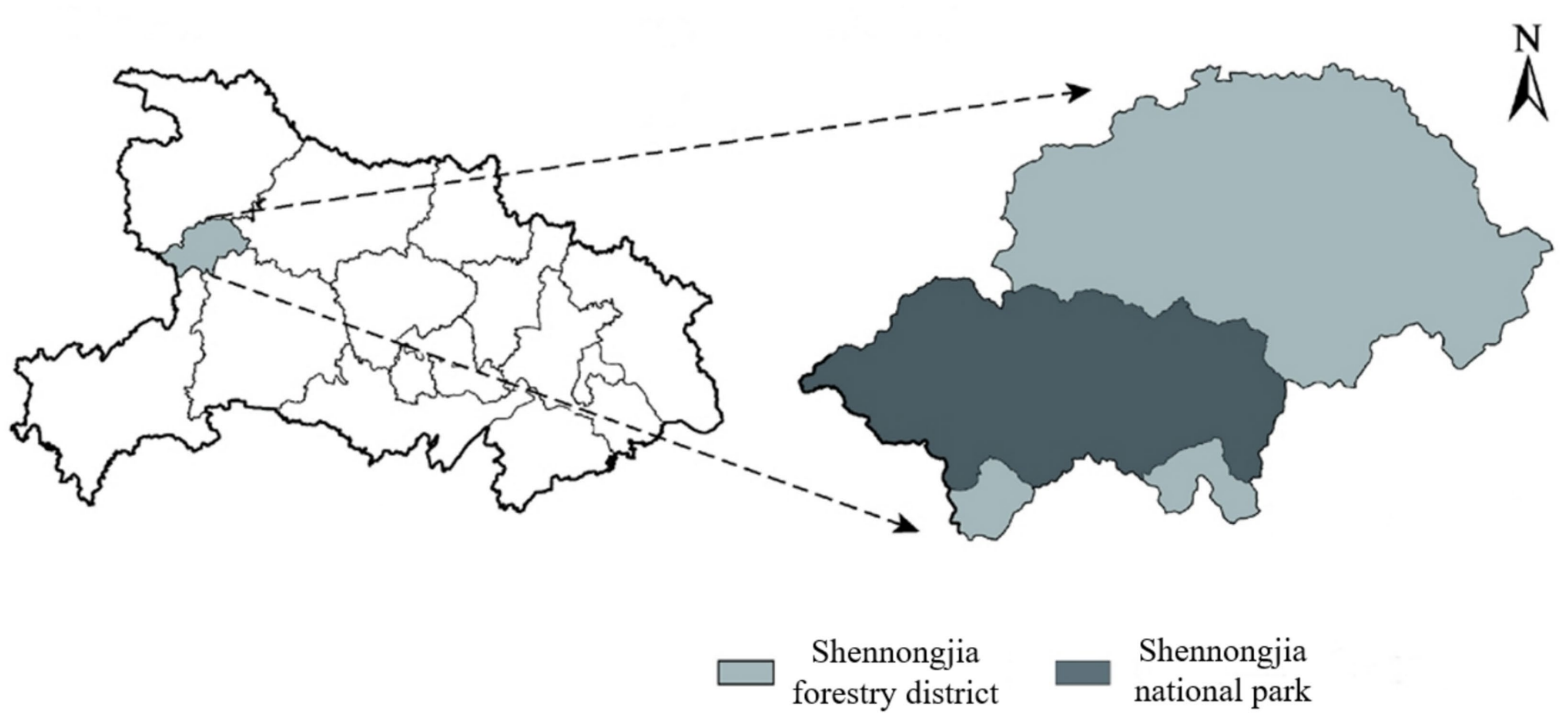
Shennongjia National Park location map.

The Shennongjia National Park pilot area is located in the Shennongjia Forest District of Hubei Province (see Figure 1), with a total area of 1,325.06 square kilometers and a permanent population of nearly 20,000 in the park. As one of the first pilot national park systems in China, Shennongjia National Park is not only a key area for global biodiversity conservation, but also a typical case of synergy between ecotourism and economic development. The pilot area covers five townships, including Muyu Township, DaJiuhu Township, Xiagu Township, Hongping Township and Songluo Township, with a total of 28 village-level units (including 23 administrative villages and five community neighborhood committees), of which 25 village-level units have permanent residents within the national park.

Before being listed as a pilot national park area in 2016, Shennongjia was already a well-known tourist destination at home and abroad, with rich natural resources and unique cultural landscapes, and a good foundation for tourism development. After being included in the pilot national park system, Shennongjia’s tourism industry has maintained a rapid growth despite the dual challenges of ecological protection and tourism development. According to statistics, the region received 18.285 million domestic and foreign tourists in 2019, realizing a tourism revenue of 6.777 billion yuan, with tourism reception ranking among the top national parks and pilot areas in the country.

The booming tourism industry in Shennongjia has injected a strong impetus to regional economic growth, while also providing a large number of employment opportunities for local residents. Many residents have gained a stable source of income through their participation in tourism-related activities (e.g., tour guiding, transportation, accommodation, food and beverage, retail, hospitality, and recreational services), and tourism has become an important means of livelihood for local residents. The practice of balancing ecological protection and tourism development in the pilot area of Shennongjia National Park provides an ideal case study to support the study of the relationship between ecotourism and economic development in national parks and its pressure on the ecological environment.

### 3.2 Variable selection and data sources

#### 3.2.1 Explained variables

The explained variable in this study is the level of economic growth in the national park. This research measures it using the logarithmic form of the Gross Domestic Product (GDP) of the Shennongjia region.

#### 3.2.2 Core explanatory variables

The core explanatory variable is the level of ecotourism development (ED) in Shennongjia National Park. Based on principles of scientific rigor, objectivity, operability, and data availability, and considering the current level of ecotourism development in China, this study constructs an evaluation system for the ecotourism development level of Shennongjia National Park from two dimensions: tourism development level and ecological development level. The specific details are shown in Table 1. The data for measuring the ecotourism development level of Shennongjia National Park are sourced from official databases, including the China Statistical Yearbook, China City Statistical Yearbook, and China Culture and Tourism Statistical Yearbook.

**Table 1:** Evaluation index system for ecotourism development level.

Primary indicator	Secondary indicator	Attribute	Explanation
Tourism development level	Passenger turnover (10,000 person-times)	Positive (+)	Reflects the scale of ecotourism activities and their contribution to regional economic growth.
	Number of tourist attractions (units)	Positive (+)	Indicates the development level of ecotourism resources and their role in promoting economic development.
	Number of tourism employees (10,000 persons)	Positive (+)	Represents the employment opportunities created by ecotourism, supporting local economic development.
	Total number of travel agencies (units)	Positive (+)	Reflects the capacity of tourism services to support ecotourism and economic growth.
Ecological development level	Investment in industrial pollution control (100 million yuan)	Positive (+)	Measures the efforts to mitigate environmental impacts, ensuring sustainable ecotourism development.
	Total agricultural output value (100 million yuan)	Positive (+)	Reflects the economic benefits of eco-friendly agricultural practices, balancing development and ecological protection.
	Total forestry output value (100 million yuan)	Positive (+)	Represents the economic and ecological value of sustainable forest resource utilization.
	Forest coverage rate (%)	Positive (+)	Directly indicates the success of ecological conservation efforts, supporting sustainable ecotourism.
	Wastewater discharge (10,000 tons)	Negative (−)	Reflects the environmental pressure from tourism and economic activities, highlighting the need for sustainable practices.
	Tourist carbon footprint (kg/person)	Negative (−)	Measures the ecological footprint of tourism and economic activities, emphasizing the importance of low-carbon development.

##### 3.2.2.1 Dependent variable: regional economic growth level

To assess the driving effect of ecotourism on regional economic growth, this study uses the Gross Domestic Product (GDP) of the Shennongjia region as a proxy variable for economic growth level. Following the standardized approach in existing literature, the natural logarithm of GDP (lnGDP) is adopted to smooth the data, mitigating heteroscedasticity and enhancing the comparability of time series data.

##### 3.2.2.2 Independent variable: ecotourism development level

This study constructs a comprehensive evaluation system for ecotourism development from two dimensions: “tourism development level” and “ecological development level” (see Table 1), aiming to comprehensively reflect the multifaceted attributes of ecotourism in Shennongjia National Park. The specific data sources cover official databases such as the China Statistical Yearbook and China Culture and Tourism Statistical Yearbook* from 2016 to 2022.

Based on the above evaluation index system, this paper adopts the entropy weight method to determine the weights of the indexes, and the calculation steps are as follows.

(1) First of all, the raw data are standardized:

Positive indicators:


(1) $$i_{xy}^{\prime }=\frac{i_{xy}-\min i_{y}}{\max i_{y}-\min i_{y}}$$

Negative indicators:


(2) $$i_{xy}^{\prime }=\frac{\max i_{y}-i_{xy}}{\max i_{y}-\min i_{y}}$$

In Equations 1, 2: $i_{xy}$ is the first *x* (*x* = 1,2,….,*t*) sample in the *y*(*y* = 1,2,…,w) indicators on the original value. $\max i_{y}$ , $\min i_{y}$ are the maximum and minimum value of the *y*-th indicator, respectively. $i_{xy}^{\prime }$ is the standardized value.

(2) Calculate the proportion value $u_{xy}$ of the *x*-th sample on the *y*-th indicator as shown in Equation 3:


(3) $$u_{xy}=\frac{i_{xy}^{\prime }}{\sum \underset{x}{\sum }\;i_{xy}^{\prime }}$$

(3) Calculate the information entropy $e_{y}$ of the *y*-th indicator as shown in Equation 4:


(4) $$e_{y}=-z\underset{x}{\sum }\;u_{xy}\ln\left(u_{xy}\right)$$

Where $z=1/\ln\left(\mathrm{wt}\right)$ and *z* > 0 such that $e_{y}$ ≥ 0.

(4) Calculate the redundancy of the information entropy of the *y*-th indicator $d_{y}$ as shown in Equation 5:


(5) $$d_{y}=1-e_{y}$$

(5) Calculate the weight $\omega_{y}$ of the *y*-th indicator as shown in Equation 6:


(6) $$\omega_{y}=d_{y}/\underset{y}{\sum }\;d_{y}$$

(6) Calculate the weighting matrix $I_{xy}$ as shown in Equation 7:


(7) $$I_{xy}=i_{xy}^{\prime }\times \omega_{y}$$

(7) Calculate the Euclidean distance $D_{x}$ :


(8) $$D_{x}^{+}=\sqrt{\sum {\left(K_{xy}-K_{y}^{*+}\right)}^{2}\;}$$

(9) $$D_{x}^{-}=\sqrt{\sum {\left(K_{xy}-K_{y}^{*-}\right)}^{2}}$$

In Equations 8, 9: $K_{xy}$ denotes the standardized score of the *y*-th index of the *x*-th sample. $K_{y}^{*+}$ and $K_{y}^{*-}$ are the positive and negative ideal solutions, respectively. $D_{x}^{+}$ and $D_{x}^{-}$ denote the Euclidean distance between the *x*-th sample and its positive and negative ideal values, respectively, which is used to measure the comprehensive superiority of the sample. The calculation of Euclidean distance is used to measure the proximity of each evaluation object to the ideal solution, and the closer the distance, the closer the performance of the evaluation object is to the ideal state.

(8) Calculate the composite score $C_{x}$ as shown in Equation 10:


(10) $$C_{x}=D_{x}^{-}/\left(D_{x}^{+}+D_{x}^{-}\right)$$

The final comprehensive evaluation score $C_{x}$ is calculated based on the ratio of the distance between the object and the positive and negative ideal solutions, and is used to rank or evaluate the overall performance of each evaluation object. The larger the value, the closer the object is to the ideal state.

#### 3.2.3 Control variables

This paper introduces human capital, infrastructure, industrial development, openness to the outside world, and government intervention as control variables to be added into the empirical study. The consideration of control variables is to try to avoid the endogenous effects caused by the omission of variables, so as to be able to ensure the reliability and validity of the measurement results. (1) Human capital (HC): the number of college students enrolled in general higher education institutions per 10,000 people is used as a proxy variable for human capital. The human capital variable is a prerequisite for economic growth. (2) Capital stock (CS): This paper uses the perpetual inventory method to estimate the capital stock, in which the depreciation rate is set at 11.85%. (3) Infrastructure (INFRA): the value of per capita road area in Shennongjia area is used to indicate the level of infrastructure. A perfect infrastructure can reduce transaction costs, promote resource flow and provide support for economic growth. (4) Industrial structure (IS): The value added of the tertiary industry as a proportion of the regional GDP is used to indicate the industrial structure. The tertiary industry (especially the service industry) is a key driver of modern economic growth, and an increase in its share helps optimize the economic structure. (5) Government Intervention (GI): The proportion of government fiscal expenditures to GDP indicates the degree of government intervention. The government regulates economic activities through fiscal expenditures, and the level of its intervention directly affects the efficiency of resource allocation and the quality of economic growth. (6) Openness to the outside world (OPEN): the proportion of the actual utilization of foreign investment to the regional GDP indicates the level of openness to the outside world. Openness to the outside world attracts foreign investment, technology and management experience, and promotes the integration of the regional economy into the global value chain.

### 3.3 Comprehensive evaluation model

In order to systematically evaluate the interactions among ecotourism, economic development and ecological environment, this study constructs a comprehensive evaluation model covering three subsystems, and calculates ETI, EDI, and EPI, respectively. The model expressions are as follows in Equation 11:


(11) $$ETI=\overset{n}{\underset{j=1}{\sum }}w_{j}^{ET}\times X_{ij}^{BT},EDI=\overset{n}{\underset{j=1}{\sum }}w_{j}^{ED}\times X_{ij}^{ED},EPI=\overset{n}{\underset{j=1}{\sum }}w_{j}^{BP}\times X_{ij}^{BP}$$

Where, *w* is the weight of the indicator calculated by entropy value method. $X_{i}$ is the value of the indicator after standardization. The superscripts ET, ED and EP correspond to the indicator sets of ecotourism, economy and ecological environment system, respectively.

### 3.4 Coupling coordination degree model

#### 3.4.1 Coupling degree model

The coupling degree is used to measure the strength of interaction among the three systems of ecotourism, economy and ecology, as shown in Equation 12:


(12) $$C=3\times \sqrt[{3}]{\frac{ETI\times EDI\times EPI}{{\left(ETI+EDI+EPI\right)}^{3}}}$$

Where the value range: *C*∈[0,1], when *C* = 1 indicates that the three systems are in optimal synergy, and *C* = 0 indicates that there is no correlation between the systems.

#### 3.4.2 Coupling coordination degree model

The degree of coupling coordination further reflects the quality of synergistic development between the systems, and the formula in Equations 13,14:


(13) $$D=\sqrt{C\times T}$$

(14) N=αETI+βEDI+γEPI

Where: *D* is the coupling coordination degree of the 2 systems; *N* is the comprehensive development index of the system. *α*, *β* are coefficients to be determined and *α* + *β* = 1. T is the comprehensive development index, reflecting the overall development of the three systems of the water. *α*, *β*,*γ* are the weight coefficients, which are determined by entropy weighting method (satisfy *α* + *β* + *γ* = 1).

In the coupled coordination degree model of ecotourism, economic development and ecological environment three systems, the setting of weight coefficients *α, β,γ* directly affects the calculation results of comprehensive development index T. Based on the theory of sustainable development, this study regards economic development and ecological environmental protection as equally important, while taking into account the driving role of ecotourism, and sets the weighting parameters as:

*α* = 0.4 (ecotourism), β = 0.3 (economic development), *γ* = 0.3 (ecological environment).

This setting reflects the core position of ecotourism in regional development and balances the dual objectives of economic growth and ecological protection.

In order to more accurately assess the synergistic development status of the three systems of ecotourism, economy and ecological environment, this study divided the coupling coordination degree *D* into 10 grades (see Table 2).

**Table 2:** Classification of coupling coordination degree.

Range	Coordination level	Description
0.100–0.199	Severe imbalance	Weak synergy; ecotourism and economic development pressure the environment.
0.200–0.299	Moderate imbalance	Limited synergy; negative ecological impacts begin to emerge.
0.300–0.399	Mild imbalance	Initial synergy forms; sustainability challenges remain.
0.400–0.499	Near imbalance	Synergy strengthens; environmental costs remain high.
0.500–0.599	Barely coordinated	Significant synergy; ecological pressure is preliminarily alleviated.
0.600–0.699	Primary coordination	Strong synergy; positive interaction with ecological protection begins.
0.700–0.799	Intermediate coordination	Synergy strengthens; negative ecological impacts are controlled.
0.800–0.899	Good coordination	Highly optimized synergy; high-level coordination achieved.
0.900–1.000	High-quality coordination	Optimal synergy; comprehensive sustainable development achieved.

On the basis of analyzing the coupled coordination state, the internal factors limiting the development of the system were further identified by comparing ETI, EDI and EPI:

If ETI > EPI and EDI > EPI; it is ecological environment lag type. It is characterized by: the speed of ecotourism and economic development exceeds the carrying capacity of the ecological environment, and the ecological environment becomes the main bottleneck of system development.

If ETI > EDI and EPI > EDI, it is economic lag type. It is characterized by a high level of ecotourism and ecological environmental protection, but the economic development is relatively lagging behind and cannot be fully converted into regional economic benefits.

If EDI > ETI and EPI > ETI, it is the ecotourism lag type. It is characterized by a high level of economic development and ecological environmental protection, but the development of ecotourism is insufficient and fails to give full play to its economic and ecological benefits.

If ETI ≈ EDI ≈ EPI, it is the synchronized development type. It is characterized by the balanced development of ecotourism, economy and ecological environment, and the synergistic effect among the systems reaches the optimal state.

### 3.5 Spatial measurement modeling

#### 3.5.1 Model selection and setting

In order to comprehensively analyze the spatial interaction of ecotourism, economic development and ecological pressure in Shennongjia National Park, this study adopts the spatial lag model (SLM), spatial error model (SEM) and spatial Durbin model (SDM) for the empirical analysis. These three models are currently the mainstream spatial measurement models. The expression of spatial lag model (SLM) is similar to the autoregressive model in time series, so it is also called spatial autoregressive model (SAR). It is mainly used to study the spatial autocorrelation of the dependent variable. The spatial error model (SEM), on the other hand, incorporates the regression term into the error term and focuses on the spatial autocorrelation of the error term. In short, SLM focuses on the autocorrelation of the dependent variable while SEM focuses on the autocorrelation of the errors. The spatial Durbin model (SDM) considers the spatial autocorrelation of both the dependent and independent variables, and the model contains two spatial weight matrices. Therefore, SLM and SEM can be considered as special forms of SDM. Due to the higher complexity of SDM, it is more applicable and more widely used in academic research as shown in Equations 15–17:

SLM:


(15) S=ρWY+Xβ+∈

SEM:


(16) S=Xβ+∈,∈=λW∈+μ

SDM:


(17) S=ρWY+Xβ+WXθ+∈

Where: ρ is the spatial autocorrelation coefficient; λ is the spatial error coefficient; β is the coefficient vector of the independent variables; θ is the coefficient vector of the spatially lagged terms of the independent variables; ϵ is the random error term; μ is the random disturbance term. *W* represents the spatial weight matrix. *WY* denotes the spatially lagged term of the dependent variable, reflecting the spillover effects of the dependent variable in space. *WX* represents the spatially lagged term of the independent variables, indicating the indirect effects of the independent variables on neighboring regions. *Wϵ* stands for the spatially lagged term of the error term.

#### 3.5.2 Spatial weight matrix construction

Two types of spatial weight matrices are used in this study. The first is the binary adjacency matrix (0–1 matrix). The second is the geographic distance matrix. Both of them can reflect the adjacency relationship between neighboring regions, in which the binary adjacency matrix is as shown in Equation 18:


(18) $$M_{xy}=\{\begin{array}{c} 1,\;\mathrm{Area}\;x\;\mathrm{is adjacent to area}\;y\;\\ 0,\;\mathrm{Area}\;x\;\mathrm{is not adjacent to area}\;y\; \end{array}$$

In addition to adjacency, spatial units can be described by distance. In spatial econometrics, this usually refers to the distance between the centers of mass or administrative centers of two regions, i.e., the geographic distance matrix. It is constructed as follows shown in Equation 19 and Table 2:


(19) $$M_{xy}=\frac{1}{{\mathrm{distance}}_{xy}}$$

Where: distance denotes the distance between the administrative centers of region *x* and region *y*. The farther the distance, the smaller the spatial correlation.

### 3.6 Spatial correlation test

To uncover the spatial distribution traits of ecotourism, economic growth, and ecological pressure within Shennongjia National Park, along with their interrelations, this research employs spatial correlation analysis. This approach encompasses both global and local spatial correlation assessments.

#### 3.6.1 Global spatial correlation test

Global spatial correlation is measured by Moran’s *I*, which is used to assess the overall spatial distribution characteristics of ecotourism, economic and ecological environmental pressure. Moran’s index is calculated as shown in Equation 20:


(20) $${\mathrm{Moran}}^{'}s\;I=\overset{\;t}{\underset{x=1}{\sum }}\;\overset{\;t}{\underset{y=1}{\sum }}\;M_{xy}\left(J_{x}-\bar{J}\right)/s^{2}\overset{\;t}{\underset{x=1}{\sum }}\;\overset{\;t}{\underset{y=1}{\sum }}\;M_{xy}$$

Where: *t* is the number of spatial units; $J_{x}$ and $J_{y}$ are the observed values of the iind and *y*-th spatial units; $\bar{J}$ is the mean value of the observed values; and $M_{xy}$ is the spatial weighting matrix, reflecting the neighborhood or distance relationship between spatial units *x* and *y*.

#### 3.6.2 Local spatial autocorrelation Moran index

The global Moran index only reflects the overall spatial correlation and cannot identify the heterogeneous characteristics of the local area. Therefore, Local Moran’s I and LISA (Local Indicators of Spatial Association) clustering diagrams were further used in this study to analyze the local spatial correlation. The Local Moran’s Index is calculated as shown in Equation 21:


(21) $$I=\frac{\left(J_{x}-\bar{J}\right)}{s^{2}}\overset{t}{\underset{y=1}{\sum }}\;M_{xy}\left(J_{x}-\bar{J}\right)$$

Where $S^{2}$ is the variance of the observations; the other variables are defined in line with the global Moran index.

## 4 Result analysis and discussion

Based on the index system and model framework constructed in the previous section, this chapter verifies the interaction of ecotourism, economic development and ecological environment pressure and its spatial effect through empirical analysis. The specific experimental process is as follows:

Spatial correlation test: analyze the spatial distribution characteristics of ETI, EDI and EPI by using global and local Moran’s index, identify the clustering patterns among regions, and visualize the spatial heterogeneity through LISA clustering diagram.

Spatial econometric modeling: the optimal model (SDM) is screened by LM test and Hausman test to quantify the direct effect and spatial spillover effect of ecotourism on economic growth, and at the same time analyze the spatial dependence of ecological and environmental pressure.

Dynamic synergy analysis: time series analysis based on the coupled coordination degree model to reveal the synergistic development trajectory of the three systems during the period of 2016–2022, and to clarify the core driving factors of the ecological and environmental lagging-type contradiction.

### 4.1 Spatial autocorrelation test

By calculating the global Moran’s Index (Moran’s I), it is found that the ETI, EDI and EPI of Shennongjia National Park all show significant spatial positive correlation (see Table 3).

**Table 3:** Global Moran’s index (2016–2022).

Year	ETI (Moran’s *I*)	EDI (Moran’s *I*)	EPI (Moran’s *I*)
2016	0.28***	0.42***	0.15**
2018	0.31***	0.45***	0.18**
2020	0.33***	0.47***	0.20**
2022	0.35***	0.49***	0.21**

***, **, indicate that *p* is significant at the 99, 95, and 90% confidence levels, respectively.

As can be seen from Table 3, the global Moran index of Shennongjia National Park is 0.28–0.35 (*p* < 0.01) on the ETI, indicating that the ecotourism activities show a spatial “high-high” or “low-low” agglomeration pattern. On the EDI: the global Moran index was 0.42–0.49 (*p* < 0.01), significantly higher than the ETI, indicating a stronger spatial dependence on the level of economic development. On EPI: the global Moran index was 0.15–0.21 (*p* < 0.05), indicating higher spatial heterogeneity of ecological pressure.

**Figure 2:**
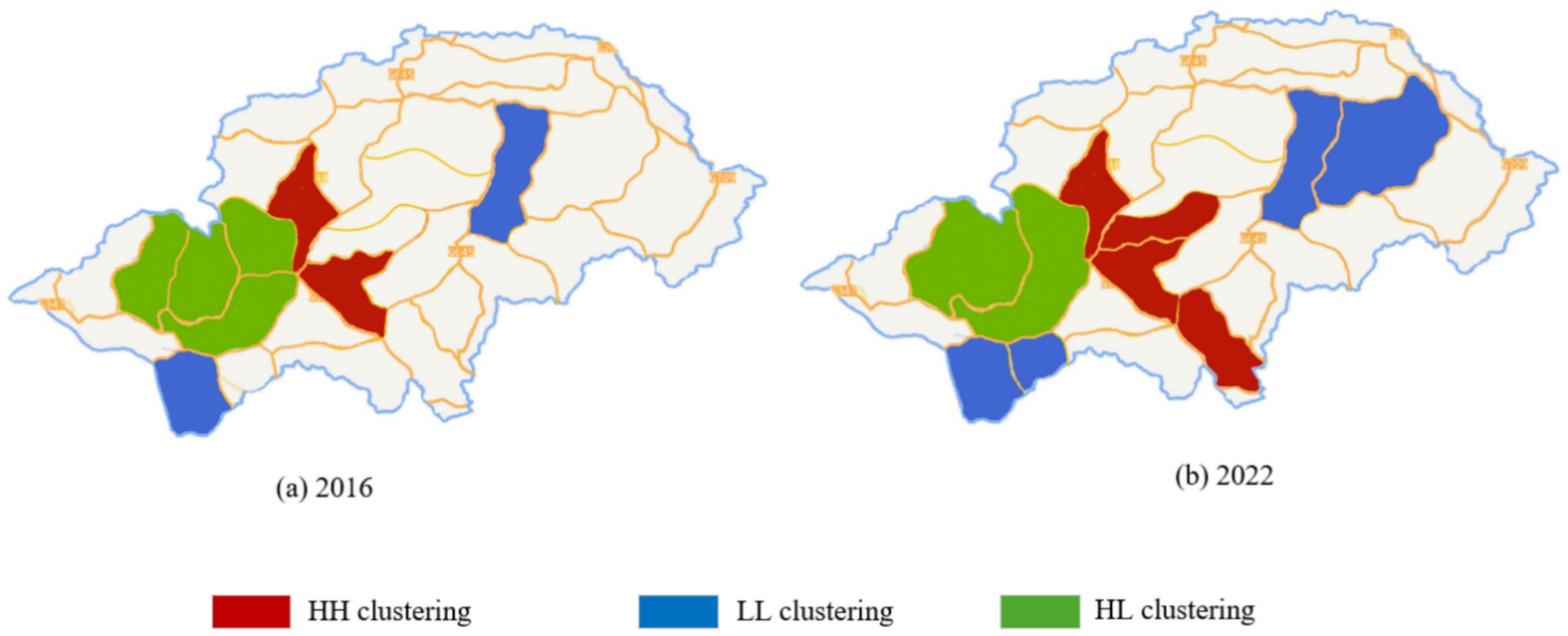
LISA cluster map of Shennongjia National Park.

Localized spatial heterogeneity was further identified by LISA clustering map (see Figure 2). High-High clustering (HH): Tourist hotspot areas such as Muyu Town and Hongping Town exhibit strong synergy between ecotourism and economic growth, but environmental pressure has simultaneously increased. Low-Low clustering (LL): Some villages in Xiagu Township and Songluo Township, constrained by limited transportation and resource scarcity, demonstrate lower levels of ecotourism and economic development, with relatively lower environmental pressure. High-Low clustering (HL): Villages around Dajiuhu Lake, restricted by wetland conservation policies that limit tourism development, represent a unique type characterized by lagging economic growth but relatively low environmental pressure.

### 4.2 Econometric model analysis

Before establishing the spatial econometric model, this paper firstly establishes the linear regression model, fixed effect model and random effect model to estimate the role of ecotourism development on economic growth without considering the spatial factors, and the results are shown in Table 4.

**Table 4:** Econometric model regression results.

Variable	OLS	Fixed effects	Random effects
ED	0.016***	0.024***	0.014***
HC	0.019***	0.026***	0.016***
CS	0.415***	0.324***	0.381***
INFRA	0.290***	0.615***	0.737***
IS	0.148***	0.201***	0.227***
GI	0.213***	0.183***	0.165***
OPEN	0.118***	0.171***	0.192***
*R* ^2^	0.692	0.635***	0.598***
*F*-value	174.56***	53.89***	141.45***
LR-value			142.34**

***, **, indicate that *p* is significant at the 99, 95, and 90% confidence levels, respectively.

Both the OLS model and the fixed effects model passed the F-test, and the LR value of the random effects model passed the 1% significance test. According to the regression coefficient results, it is found that the regression coefficients of the core explanatory variable ecotourism development level are significantly positive in all the three models mentioned above, which indicates that ecotourism development has a positive promotion effect on urban economic growth. From the results of spatial autocorrelation test, it can be found that the level of ecotourism development and economic growth have positive spatial correlation. In order to deeply analyze the spatial effect of ecotourism development level on economic growth, this paper selects the 0–1 adjacency matrix to establish a spatial econometric model for further research. First of all, the model is subjected to LM test and Robust_LM test to determine its specific form, and the test results are shown in Table 5.

**Table 5:** LM test.

Test indicator	Statistic	*p*-value
SEM-LM	120.345	0.001
SEM-RobustLM	5.678	0
SLM-LM	160.789	0.002
SLM-RobustLM	60.123	0

According to the results in Table 5, the *p*-values of the LM test for both SEM and SLM pass the test of significance, and therefore the original hypothesis is rejected. The results of the Robust_LM test for SEM and SLM also pass the test of significance, and the original hypothesis is likewise rejected. In order to examine the applicability of the spatial Durbin model, this paper also conducted the LR test and Wald test results as shown in Table 6. As can be seen from the results in Table 6, both the LR and Wald tests pass the significance test, indicating the applicability of the spatial Durbin model. The Hausman test determines whether to choose fixed effects or random effects, and in this paper, the Hausman test was conducted with a statistic of 112.691, *p* < 0.001.Therefore, the spatial Durbin fixed effects model was used in this paper for the study.

**Table 6:** LR test and Wald test.

Test indicator	Statistic	*p*-value
SEM-LR	58.45	0.000
SEM-Wald	38.76	0.000
SLM-LR	50.32	0.000
SLM-Wald	28.91	0.000

Through LM test, Robust LM test, LR test and Wald test (Tables 5, 6), SDM was determined to be the optimal model. Therefore, this paper adopts SDM to empirically analyze the spatial interaction of ecotourism, economic development and ecological environment pressure in Shennongjia National Park. The results of Hausman test (statistic = 112.691, *p* < 0.001) support the fixed effect model.

### 4.3 Analysis of regression results

On the basis of the diagnostic test of the spatial econometric model, the spatial econometric model applicable to the sample data selected for the study in this paper is established through spatial regression. In this section, the regression results will be analyzed using the ecotourism development level and economic growth of Shennongjia National Park as well as other control variables data, and the results are shown in Table 7.

**Table 7:** Regression results of spatial econometric model.

Variable	SLM	SEM	SDM
ETI	0.62***	0.59***	0.68***
EPI	−0.18**	−0.21**	−0.23**
Spatial spillover effect(ρ)	0.15*	—	0.19*
Log-likelihood	432.5	428.7	454.8
R^2^	0.571	0.563	0.595

***, **, * indicate that *p* is significant at the 99, 95, and 90% confidence levels, respectively.

From the regression results in Table 7, it can be found that the regression results of ecotourism development on the city’s economic growth in the SLM model, SEM model and SDM model are all significantly positive. Among them, ETI: the coefficient is 0.68 (*p* < 0.01), indicating that for every 1 unit of ecotourism enhancement, the regional economic growth rate increases by 0.68%. The results verify that the development of regional ecotourism can promote economic growth. EPI: the coefficient is −0.23 (*p* < 0.05), which verifies the negative impact of tourism activities on the ecological environment. Spatial spillover effect: ecotourism development in neighboring regions generates a positive spillover on economic growth in the region through resource sharing (*ρ* = 0.19, *p* < 0.1).

### 4.4 Dynamic analysis of the coupled and coordinated development level of tourism economy and ecological environment

**Figure 3:**
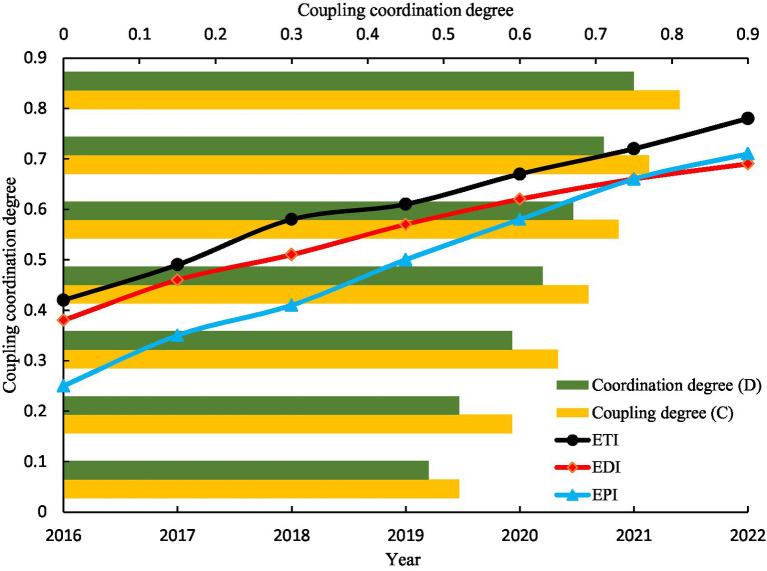
Evolutionary trend of the coupling coordination degree of the three indicators in Shennongjia National Park (2016–2022).

By constructing a three-system coupled coordination degree model, we calculate the ETI, EDI and EPI of Shennongjia National Park from 2016 to 2022 and analyze its synergistic evolution trend. The experimental results are shown in Table 8 and Figure 3.

**Table 8:** Evaluation results of coupled coordination degree of Shennongjia National Park (2016–2022).

Year	ETI	EDI	EPI	Coupling degree (C)	Coordination degree (D)	Coordination level	System type
2016	0.42	0.38	0.25	0.52	0.48	Near imbalance	Economic lagging type
2018	0.58	0.51	0.41	0.65	0.59	Barely coordinated	Ecological environment lagging type
2020	0.67	0.62	0.58	0.73	0.67	Primary coordination	Ecological environment lagging type
2022	0.78	0.69	0.71	0.81	0.75	Intermediate coordination	Synchronized development type (partial)

From Table 8 and Figure 3, the dynamic characteristics of the comprehensive development indices can be summarized as follows: ETI: Increased from 0.42 in 2016 to 0.78 in 2022, with an average annual growth rate of 12.3%. This growth is primarily attributed to the construction of smart scenic areas and the development of ecological products. EDI: Rose from 0.38 to 0.69, indicating the significant role of tourism in driving regional economic growth. EPI: Increased from 0.25 to 0.71, with localized areas (e.g., Shennongding Scenic Area) experiencing vegetation degradation and water quality decline due to tourist overload.

The evolution trends of coupling coordination are as follows: Coupling degree (C): Improved from 0.52 to 0.81, indicating a significant enhancement in the interaction among the three systems. Coordination degree (D): Increased from 0.48 (near imbalance) to 0.75 (intermediate coordination), yet it has not reached the level of high-quality coordination (*D* > 0.9).

## 5 Conclusion and policy recommendations

This study reveals the complex interactive relationship between ecotourism, economic development and ecological environment through empirical analysis of Shennongjia National Park. The findings indicate that ecotourism significantly contributes to regional economic growth but also increases ecological pressure. Based on the empirical results, this study proposes the following development pathways and strategies to achieve synergistic development of ecotourism, economic growth, and ecological conservation, providing scientific insights for the sustainable development of global protected areas.

First, optimize spatial layout and functional zoning by dividing Shennongjia National Park into core protection zones, buffer zones, and peripheral zones. Implement strict control in core zones, allowing only scientific research and monitoring, and establish electronic fences and real-time monitoring systems. In buffer zones, conduct low-intensity ecotourism activities such as ecological education and hiking, while setting visitor capacity limits and adopting reservation systems. In peripheral zones, develop tourism service facilities like accommodation, dining, and transportation, creating a “peripheral service, core protection” spatial pattern. Encourage community participation in tourism services and provide employment training and financial support.

Second, build a digital management platform using IoT, remote sensing, and big data technologies for real-time monitoring and early warning. Deploy sensor networks to monitor key ecological indicators and develop intelligent warning systems. Guide tourists toward environmentally friendly behavior through smart terminals and mobile applications, such as a “Smart Scenic Area” app offering e-guides, reservation services, and eco-friendly tips. Establish a dynamic database for ecotourism and economic development to support scientific decision-making and policy optimization, and regularly publish ecotourism development reports to evaluate conservation and development outcomes.

In addition, this study also suggests constructing a ‘Carbon Sink Tourism Product Development System’, linking the Tourist Carbon Footprint (TCF) to the carbon sink trading mechanism. For example, by attaching a ‘Carbon Neutral Fund’ to the entrance ticket, guiding tourists to voluntarily participate in carbon compensation (e.g., withdrawing 1 Yuan per ticket for forest carbon sink projects), and realizing the transparency of carbon sink trading through blockchain technology. At the same time, explore ecological compensation models linked to the ‘dual-carbon’ goal, such as incorporating the incremental carbon sinks of national parks into the local carbon emissions trading market, forming a closed-loop mechanism of ‘protection-trading-reinvestment’.

While this study provides valuable insights into the interactions between ecotourism, economic development, and ecological environment in Shennongjia National Park, it has certain limitations that warrant further exploration. One key limitation is the reliance on quantitative data from official statistical sources, which may not fully capture the socio-economic and cultural impacts of ecotourism on local communities. Future research could integrate qualitative methods, such as interviews and surveys with local residents and stakeholders, to provide a more comprehensive understanding of these dimensions. This would enhance the robustness of the findings and offer deeper insights into the human aspects of ecotourism development.

## Data Availability

The original contributions presented in the study are included in the article/supplementary material, further inquiries can be directed to the corresponding author.
